# Efficacy and Safety of Bempedoic Acid to Prevent Cardiovascular Events in Individuals at Risk of Cardiovascular Diseases: A Meta-Analysis of Randomized-Control Trials

**DOI:** 10.7759/cureus.38662

**Published:** 2023-05-07

**Authors:** Gautham Varun Krishna Mohan, Venkata Sai Harshabhargav Chenna, Gayathri Tirumandyam, Abdur Rehman Mian, Atif Rashid, Faraz Saleem

**Affiliations:** 1 Internal Medicine, Tirunelveli Medical College, Tirunelveli, IND; 2 Medicine, University of Perpetual Help System Dalta, Las Pinas, PHL; 3 Internal Medicine, Siddhartha Medical College, Dr NTR University of Health Sciences, Gunadala, IND; 4 Internal Medicine, Caribbean Medical University, Willemstad, CUW; 5 School of Medicine, Caribbean Medical University, Rosemont, USA; 6 Internal Medicine, California Institute of Behavioral Neurosciences & Psychology, Fairfield, USA; 7 Internal Medicine, Akhtar Saeed Medical and Dental College, Lahore, PAK

**Keywords:** efficacy, meta-analysis, heart diseases, cardio vascular disease, bempedoic acid

## Abstract

The purpose of this study was to evaluate the effectiveness and safety of bempedoic acid in preventing cardiovascular events among high-risk patients. We conducted a meta-analysis following the Preferred Reporting Items for Systematic Reviews and Meta-Analyses (PRISMA) guidelines. Two independent researchers carried out online database searches on Medline, the Cochrane Library of Clinical Trials, and EMBASE until April 15, 2023, using search terms such as "bempedoic acid," "cardiovascular outcomes," and "randomized controlled trial." We also utilized medical subject heading (MeSH) terms and Boolean algebra operators to refine our search. We included articles that compared cardiovascular outcomes between patients receiving bempedoic acid and those receiving a placebo. The primary outcome assessed was major adverse cardiovascular events (MACE), defined as a composite of cardiovascular death, myocardial infarction, nonfatal stroke, hospitalization for unstable angina, and coronary revascularization. The meta-analysis included three randomized controlled trials with a total of 16,978 patients. The use of bempedoic acid was associated with a significant reduction in major adverse cardiovascular events. Individual analyses reported a low risk of myocardial infarction, coronary revascularization, and hospitalization due to unstable angina in patients receiving bempedoic acid. Furthermore, our meta-analysis found that bempedoic acid is a safe treatment option, as there was no significant difference between the bempedoic acid and placebo groups in terms of adverse events and serious adverse events. Our findings support the use of bempedoic acid as a promising treatment option for high-risk cardiovascular patients. However, since our meta-analysis included a limited number of studies with short follow-up periods, larger studies are necessary to provide more definitive evidence.

## Introduction and background

Hypercholesterolemia is defined as an excess of cholesterol in the bloodstream. It is one of the significant risk factors for cardiovascular disease, which is the leading cause of death globally [1]. According to the current guidelines from the American College of Cardiology/American Heart Association, patients at high risk of cardiovascular disease should aim to reduce their low-density lipoprotein cholesterol (LDL-C) levels by at least 50% [2]. The use of drugs that lower lipid levels, especially statins, has greatly decreased the incidence of cardiovascular disease in the last 30 years [3]. However, statins alone may not be enough to achieve the desired reduction in LDL-C levels [4-5]. Unfortunately, between 7% and 29% of people may be intolerant to statins, often experiencing muscle-related side effects. When someone cannot tolerate statins, their cholesterol levels may remain uncontrolled, which increases their risk of cardiovascular disease [6]. To address this problem and reduce cardiovascular risk in these patients, the 2018 multi-society guidelines recommend the use of non-statin drugs in addition to statins [7].

Bempedoic acid is a new type of drug that inhibits ATP-citrate lyase, an enzyme involved in the production of cholesterol. This enzyme is located upstream of β-hydroxy β-methylglutaryl-coenzyme A, which is also targeted by statins [8]. However, bempedoic acid is a prodrug, which means it needs to be activated by an enzyme called very long-chain acyl-CoA synthetase-1. This enzyme is not found in skeletal muscle, which may help prevent the muscle-related side effects associated with statins. So, while bempedoic acid affects the same pathway as statins, it may have a different mechanism of action and side-effect profile [8].

Reaching the LDL-C goal is crucial for individuals who have atherosclerotic cardiovascular disease (ASCVD) or are at high risk of MACE. The FOURIER trial analysis, which spanned over 2.2 years, showed that reducing LDL-C levels with evolocumab in conjunction with statin therapy provided additional benefits in terms of relative risk reduction of major adverse cardiovascular events (MACE). The results indicated that achieving a lower LDL-C level within four weeks did not pose any safety concerns [9]. Bempedoic acid was approved by the Food and Drug Administration (FDA) in 2020 for use in adults who require additional lowering of low-density lipoprotein cholesterol. The European Medicines Agency (EMA) has also recommended its approval for the treatment of adults with primary hypercholesterolemia and mixed dyslipidemia [10]. Many studies have emphasized bempedoic acid’s capacities for lowering lipid levels, but not many trials have focused on cardiovascular outcomes [11]. Therefore, we are conducting this meta-analysis to conduct a pooled analysis of available RCTs to assess the efficacy and safety of bempedoic acid in preventing cardiovascular events in patients at high risk of cardiovascular diseases.

## Review

Methodology

The present meta-analysis was conducted according to the Preferred Reporting Items for Systematic Reviews and Meta-Analyses (PRISMA) guidelines.

Search Strategy and Study Selection

Two researchers independently conducted online database searches of Medline, the Cochrane Library of Clinical Trials, and EMBASE until April 15, 2023, to identify articles that compared cardiovascular outcomes in patients receiving bempedoic acid and placebo. The key search terms used were "bempedoic acid," "cardiovascular outcomes," and "randomized controlled trial." Medical subject heading (MeSH) terms were also used, along with Boolean algebra operators, to further refine the search. Relevant clinical trial registries, such as ClinicalTrials.gov, were also searched to identify any recently completed studies or ongoing trials whose results were available. The reference lists of all included studies were manually searched, and relevant articles were added to the study selection process.

All obtained references were imported into the EndNote X9 library. After removing duplicates, first-level screening was done using titles and abstracts. The full text of selected articles was retrieved, and a detailed assessment was done using pre-defined inclusion and exclusion criteria. Any disagreements between the two researchers in the search strategy and study selection process were resolved by consensus.

Eligibility Criteria

We included randomized controlled trials available in full-text and English language assessing cardiovascular outcomes in patients receiving bempedoic acid, either alone or in combination with other drugs or interventions. We included studies with a follow-up period of at least 12 months. We excluded non-randomized studies, reviews, case reports, and case series. We also excluded studies that did not report the primary outcome, which was major adverse cardiovascular events (MACE). There were no restrictions placed on the year of publication or sample size.

Data Abstraction, Quality Assessment and Outcomes

Data from the included studies were extracted using piloted forms developed on Microsoft Excel (Redmond, USA). The extracted data included the author's name, year of publication, region, sample size, follow-up duration, participants' characteristics, and outcome measures. Two authors extracted the data, cross-checked it, and then entered it into RevMan for statistical analysis. The primary outcome assessed was major adverse cardiovascular events (MACE), defined as a composite of cardiovascular death, myocardial infarction, nonfatal stroke, hospitalization for unstable angina, and coronary revascularization. These outcomes were also assessed separately. Secondary outcomes included a change in LDL-C from baseline in percentage. For safety, adverse events and serious adverse events (SAEs) were analyzed separately. The quality of eligible studies was assessed using the Cochrane risk of bias assessment tool. A total of seven domains were assessed as part of the quality assessment. Each domain was graded as either low, high, or unclear.

Statistical Analysis

Statistical analysis was performed using RevMan (Version 5.4.1; The Cochrane Collaboration, London, United Kingdom). Since all outcome variables were dichotomous, we used the Mantel-Haenszel method, and the risk ratio (RR) was reported along with a 95% confidence interval (CI). For continuous outcomes, we reported the mean difference (MD) with a 95% CI. Statistical significance testing was two-sided, and P < 0.05 was considered statistically significant. Heterogeneity among the study results was assessed using the I-square test, with I-square values greater than 50% being considered significant for heterogeneity.

Results

**Figure 1:**
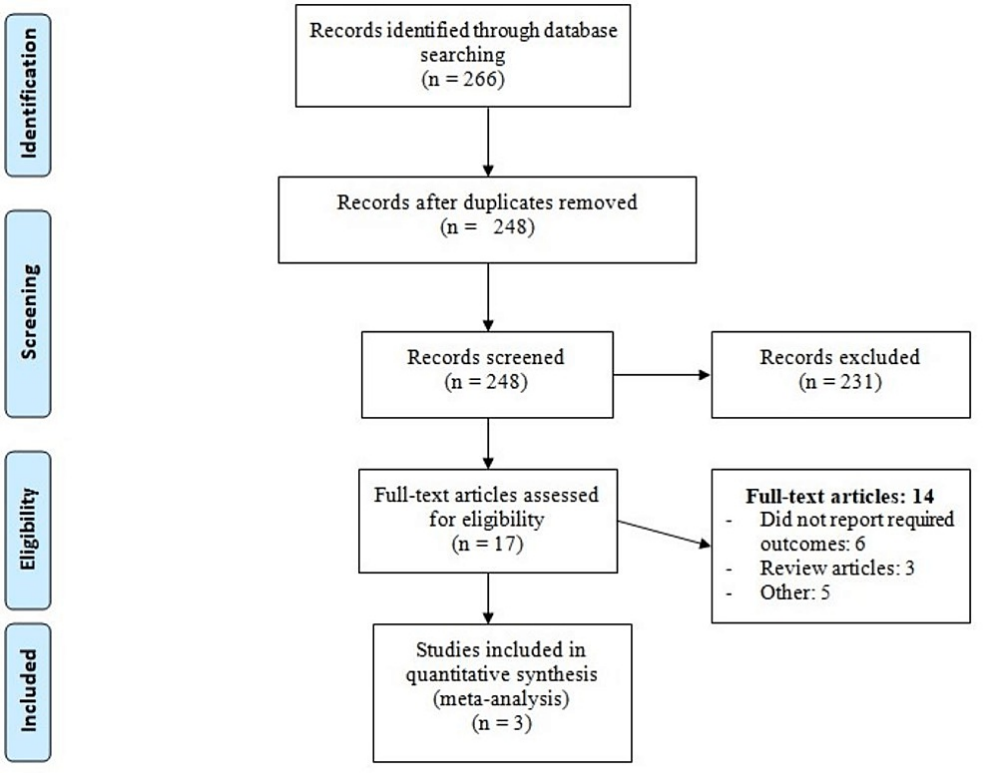
PRISMA flowchart of selection of studies

**Figure 2:**
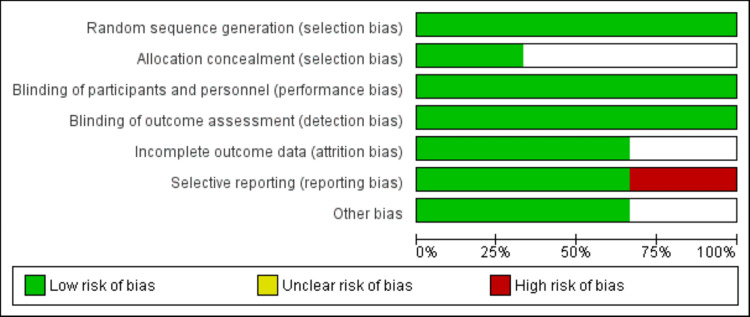
Quality Assessment

The PRISMA flow chart showing the study selection process is presented in Figure 1. Of the 266 studies initially identified, 18 were duplicates. On the first-level screening, we excluded 231 records. We obtained the full texts of 17 studies, and a detailed assessment was done to identify whether they met the inclusion criteria or not. Finally, three RCTs were included in the final analysis, enrolling 16978 patients. Table 1 presents the characteristics of the included studies. The sample size of individual studies ranged from 779 to 13970. The follow-up of individual studies ranged from 12 months to 60 months. Figure 2 shows the quality assessment of the included studies. 

**Table 1:** Characteristics of included studies

Author Name	Year	Population	Groups	Sample Size	Follow-up	Age (Years)	Males (%)	Statin at baseline (%)
Goldberg et al [12]	2019	Maximally Tolerated Statins	Bempedoic Acid	522	12 Months	64.4	63.7	100
			Placebo	257				
Nissen et al [13]	2023	Statin intolerant patients	Bempedoic Acid	6992	60 Months	65.5	51.8	22.7
			Placebo	6978				
Ray et al [14]	2019	Maximally Tolerated Statins	Bempedoic Acid	1487	12 Months	66.3	68.9	100
			Placebo	742				

Major Adverse Cardiovascular Events (MACE)

**Figure 3:**
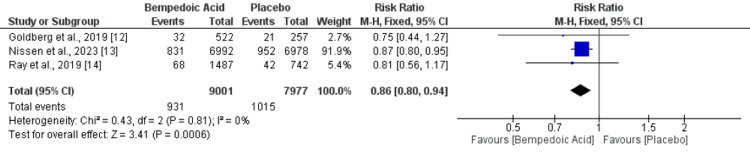
Effect of bempedoic acid on MACE Sources: References [12-14]

Three studies assessed the impact of bempedoic acid on MACE in a total of 9001 patients in the bempedoic acid group and 7977 patients in the placebo group. Overall, the pooled incidence of MACE was 11.5%, and the risk of MACE was significantly lower in patients treated with bempedoic acid compared to the placebo group (RR: 0.86, 95% CI: 0.80-0.94), as shown in Figure 3. No significant heterogeneity was reported among the study results (I-square: 0%).

Table 2 shows the results of individual MACE events. We found no significant difference between the two groups in terms of cardiovascular death and stroke. However, the risk of myocardial infarction, coronary revascularization, and hospitalization due to unstable angina was significantly lower in patients receiving bempedoic acid compared to patients randomized to the placebo group. No significant heterogeneity was reported in any of the individual outcomes.

**Table 2:** Results of individuals cardiovascular events RR: Risk ratio; CI: Confidence interval * Significant at p-value<0.05

Outcome	Number of Studies	RR (95% CI)	I-square
Cardiovascular Death	3	1.05 (0.89-1.24)	0%
Myocardial Infarction	3	0.76 (0.65-0.89)*	26%
Stroke	3	0.98 (0.78-1.24)	0%
Coronary Revascularization	3	0.81 (0.72-0.91)*	0%
Hospitalization due to unstable angina	3	0.69 (0.54-0.88)*	0%

Change in LDL-C (%)

**Figure 4:**
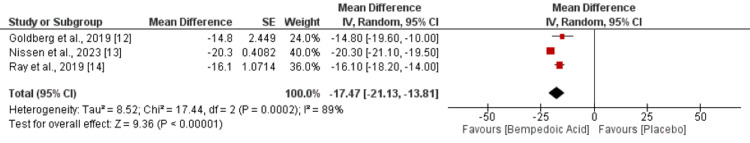
Effect of bempedoic acid on change in LDL-C from baseline (%) Sources: References [12-14]

Three studies assessed the percent change in LDL-C from baseline. Pooled analysis showed that the reduction in LDL-C was significantly greater in bempedoic acid treated patients compared to placebo-treated patients (MD: -17.47, 95% CI: -21.13, -13.81), as shown in Figure 4. High heterogeneity was reported among the study results (I-square: 89%).

Safety Analysis

**Figure 5:**
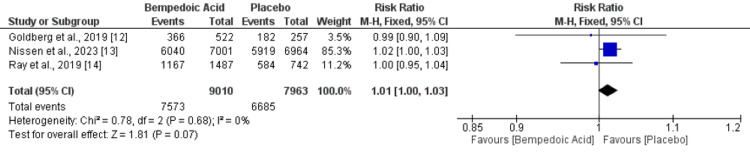
Comparison of adverse events between bemepdoic acid and placebo Sources: References [12-14]

**Figure 6:**
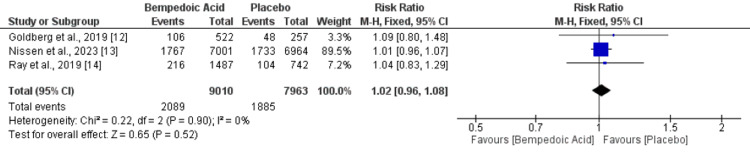
Comparison of SAE between bemepdoic acid and placebo Sources: References [12-14]

Three studies assessed adverse events and SAEs between patients randomized to bempedoic acid and placebo. No significant difference was reported between the two groups in terms of adverse events (RR: 1.01, 95% CI: 1.00-1.03), as shown in Figure 5. No heterogeneity was reported among the study results (I-square: 0%). Additionally, the risk of SAE was also similar in both groups (RR: 1.02, 95% CI: 0.96-1.08). No heterogeneity was reported among the study results (I-square: 0%), as shown in Figure 6.

Discussion

In this meta-analysis of three RCTs enrolling 16978 patients, the use of bempedoic acid was associated with a significant reduction in major adverse cardiovascular events. Individual analyses reported a low risk of myocardial infarction, coronary revascularization, and hospitalization due to unstable angina in patients receiving bempedoic acid. Moreover, the use of bempedoic acid is safe, as no significant difference between the two groups was reported in the present meta-analysis in terms of adverse events and serious adverse events (SAEs). A previous meta-analysis assessed the effeect of bempedoic acid on the reduction of low-density cholesterol (LDL) levels [15] and included five studies with a total of 625 patients with hypercholesterolemia. The study concluded that the reduction of LDL was significantly greater in patients receiving bempedoic acid without adverse events.

This study included a recently conducted large RCT that was particularly designed to assess the impact of bempedoic acid on cardiovascular illness in patients with statin intolerance [13]. This study included a large number of patients compared to the other two studies. The study provided evidence of cardiovascular benefit from the utilization of bempedoic acid in patients who are at high risk of cardiovascular illness. The lower incidence of cardiovascular events in patients receiving bempedoic acid reported in our meta-analysis is related to lower LDL cholesterol levels. Nonetheless, it differs from other drugs that lower LDL cholesterol because it is a prodrug that necessitates the activation of an enzyme called very long-chain acyl-CoA synthetase 1, which is primarily found in the liver. This characteristic of bempedoic acid may help prevent muscle-related side effects reported by certain individuals taking statins [16-17].

The study conducted by Nissen et al. [13] reported that six months of treatment with bempedoic acid led to a 21.6% decrease in the high-sensitivity C-reactive protein level compared to the placebo. Moreover, unlike statins, it did not cause a rise in glycated hemoglobin levels or the incidence of new-onset diabetes [18]. Elevated levels of C-reactive protein have been established as a reliable and consistent risk marker for preventing cardiovascular disease, both in primary and secondary cases [19]. In order to target C-reactive protein, medical professionals have the option to utilize non-statin medications like ezetimibe and bempedoic acid in conjunction with statins. However, it is important to note that PCSK9i drugs do not have an effect on C-reactive protein levels, regardless of the type of PCSK9 monoclonal antibody used [20]. Therefore, bempedoic acid can be a viable option for effectively reducing C-reactive protein when used alongside statins. Furthermore, bempedoic acid has the added benefit of not increasing the risk of developing or worsening diabetes and may even lead to a slight decrease in HbA1c levels, making it potentially advantageous in clinical settings [21]. However, further studies are needed to assess whether the decrease in C-reactive protein levels with bempedoic acid contributed to the benefits.

Future Research

Based on the results of this meta-analysis, further clinical trials are needed to explore in detail the potential cardiovascular benefits of bempedoic acid. Moreover, it remains unclear whether specific patient subgroups may benefit more from this treatment. Extended follow-up periods can be valuable in gathering essential data on the safety, tolerability, and long-term effectiveness of bempedoic acid in preventing cardiovascular outcomes. Exploring alternative treatments other than statins for preventing hyperlipidemia in specific patient groups who cannot tolerate statins is an ongoing research area. Healthcare providers should continue to consult established guidelines when selecting initial drug therapy for patients with hyperlipidemia.

Study Limitations

Meta-analysis is one feasible way to assess the clinical safety and efficacy of bempedoic acid; however, it has certain limitations. First, the present meta-analysis included only three trials, and two of the included studies explored cardiovascular outcomes as secondary or exploratory outcomes. Second, two studies followed patients for only 12 months, which may lead to imprecise effect estimates, resulting in low to moderate certainty of the consistency of estimated and true effects. Additionally, the heterogeneity in study co-medications, such as no statin versus maximally tolerated statin and additional ezetimibe, is also a limitation. We were not able to perform subgroup analysis based on statin intolerance and maximally tolerated statins due to the limited number of studies. We did not have individual-level data; therefore, we were not able to assess the risk of cardiovascular outcome in different groups of patients. Therefore, the results of the current meta-analysis are exploratory and should be interpreted with caution. Future randomized controlled trials with large sample sizes and longer follow-up periods are necessary to recommend treatment with bempedoic acid. It is also important to understand the impact of bempedoic acid in detail on statin intolerance and maximally tolerated statin patients separately. 

## Conclusions

In conclusion, our meta-analysis of three RCTs demonstrated that bempedoic acid is associated with a significant reduction in major adverse cardiovascular events, particularly the risk of myocardial infarction, coronary revascularization, and hospitalization due to unstable angina. The use of bempedoic acid was found to be safe, with no significant difference in adverse events or serious adverse events between the bempedoic acid and placebo groups. Therefore, bempedoic acid could be a viable option to effectively reduce LDL-C. Overall, our findings support the use of bempedoic acid as a promising treatment option for patients at high risk of cardiovascular illness. However, considering the small number of studies, most of which followed participants for a short period of time, large studies are required to confirm the findings that can help the development of recommendations on the use of bempedoic acid to reduce LDL-C and prevent cardiovascular events.
